# Mast Cells in the Solid Tumor Microenvironment: Multiple Roles and Targeted Therapeutic Potential

**DOI:** 10.32604/or.2025.069703

**Published:** 2025-11-27

**Authors:** Chenglu Lu, Huiting Zhang, Ujjal K. Bhawal, Lei Wang, Jingwu Li, Pangzhou Chen, Lewei Zhu

**Affiliations:** 1 Department of Pathology, Hebei Key Laboratory of Molecular Oncology, The Cancer Institute, Tangshan People’s Hospital, Tangshan, 063001, China; 2 Department of Oncology, State Key Laboratory of Oncology in South China, Guangdong Provincial Clinical Research Center for Cancer, Sun Yat-Sen University Cancer Center, Guangzhou, 510060, China; 3 Center for Global Health Research, Saveetha Institute of Medical and Technical Sciences, Saveetha Medical College and Hospitals, Saveetha University, Chennai, 600077, India; 4 Research Institute of Oral Science, Nihon University School of Dentistry at Matsudo, Chiba, 271-8587, Japan; 5 Department of Breast Surgery, The Sixth Affiliated Hospital, School of Medicine, South China University of Technology, Foshan, 528000, China; 6 The First People’s Hospital of Foshan (The Affiliated Foshan Hospital of Southern University of Science and Technology), School of Medicine, Southern University of Science and Technology, Foshan, 528000, China

**Keywords:** Tumor microenvironment, mast cell, mast cell activation, immunomodulatory, cell communication, targeted therapy

## Abstract

The tumor microenvironment (TME) is a complex network composed of non-tumor cells, extracellular matrix, blood vessels, and various molecular signals that surround and profoundly influence tumor progression. As one of the key immune effector cells within the TME, mast cells (MCs) exhibit functional complexity, and their specific roles remain widely debated. Depending on the cancer type, spatial distribution, and interactions with other TME components, MCs can demonstrate dual regulatory capabilities—either promoting or inhibiting tumor growth. This characteristic has made them an important focus in current tumor immunology research. This review aims to systematically review the current understanding of MCs in the TME, with emphasis on their characteristics and functional differences across various tumor types, pathological status, and species. In recent years, advances in the understanding of MC markers, activation mechanisms, and biological functions have made targeting specific MC subsets an emerging therapeutic strategy. By comprehensively examining the origin, activation mechanisms, cellular interactions, and therapeutic regulation of MCs, this review provides new perspectives and a basis for future directions in tumor research and treatment.

## 1 Introduction

The tumor microenvironment (TME), comprising tumor cells, non-tumor stromal cells, the extracellular matrix (ECM), vasculature, and diverse molecular signals, plays a crucial role in tumorigenesis and progression [1]. Tumors are frequently classified as “hot” or “cold” based on the extent of immune cell infiltration, a key determinant of anti-tumor immunity and therapeutic response. Mast cells (MCs), first described by Paul Ehrlich in 1879 [2], are key innate immune sentinels predominantly resident in barrier tissues like skin and mucosa, where they are vital mediators of allergic and inflammatory responses [3].

Interestingly, beyond their classical roles, MCs exhibit complex and multifaceted biological functions within TME [4]. Numerous studies have identified the pivotal role of MCs in the TME. MCs significantly influence tumor progression through diverse mechanisms, impacting tumor growth, angiogenesis, immune regulation, and even antigen presentation [5]. Recognized primarily for their role in hypersensitivity, MCs are now understood as critical modulators of the TME [6]. Their activity within the TME can exert either pro-tumorigenic or anti-tumorigenic effects, contingent upon the specific microenvironment and the profile of mediators they release.

Consequently, MCs contribute significantly to both innate and adaptive immune responses within the TME, facilitating antigen presentation and modulating T cell activity. Furthermore, the diverse array of bioactive molecules released by MCs are strongly implicated in tumor initiation and progression [7].

However, current research delineating MCs’ functions within the TME reveals considerable complexity and diversity, with recent studies highlighting divergent roles across different cancer types [8]. Comprehensive synthesis and critical examination of MCs’ contributions to the TME are therefore warranted. Given that immunotherapy, while promising, yields variable patient responses, targeting MCs emerges as a potential therapeutic strategy. Summarizing existing MC-targeting agents and clinical trials could inform future research directions. Understanding the specific functions of MCs within distinct tumor types is essential to rationally develop novel MC-targeted therapies that may enhance cancer treatment efficacy. Future research must prioritize elucidating the precise mechanisms of MCs’ action within the TME to translate these insights into effective therapeutic interventions.

## 2 Origin of MCs

MCs, as key components of the innate immune system, are distributed across various organs [9]. In adults, most hematopoietic stem cells (HSCs) and MC progenitors originate from the bone marrow (BM).

Following their egress from the BM, these cells enter the bloodstream, migrating to tissues, where they undergo maturation into functional MCs under the influence of stem cell factor (SCF) [10]. In the BM, few HSCs differentiate into MCs because of the influence of cytokines. During embryonic and fetal development, mesenchymal stem cells from the yolk sac develop into pluripotent HSCs, which circulate and infiltrate organs, including the liver, BM, and skin [11].

The phenotypic traits of MC-committed progenitor cells in mice and humans include the expression of CD34, c-KIT (CD117), IL-3R, and CD13, typically accompanied by low FcεRI levels [12]. As MCs develop, their precursors lose CD34 and IL-3R alpha (CD123) expression while increasing FcεRI and c-KIT expression [13]. Notably, these progenitor cells possess adhesion molecules that enable migration to peripheral organs and tissues.

## 3 Activation of MCs

The activation or inhibition of MCs is modulated by a range of internal and external factors that interact with diverse receptors on the MC membrane. These receptors can be categorized based on the nature of their response to ligand–receptor binding: biphasic and monophasic reactive receptors [14,15].

### 3.1 Biphasic Reactive Receptors

Biphasic Reaction Process: Upon the binding of a biphasic reactive receptor to its specific ligand, a MCs initiate a biphasic reaction. The initial phase is characterized by rapid degranulation, during which MCs release pre-synthesized bioactive substances. In the subsequent phase, MCs engage in *de novo* synthesis and release of bioactive substances [14]. Fc epsilon RI (FcεRI) is a member of the multichain immune recognition receptor (MIRR) family and is a well-characterized example of a receptor that initiates biphasic reaction. On the surfaces of MCs, FcεRI exists as a tetrameric complex composed of an α subunit, a β subunit, and a dimer of γ subunits [16]. Upon cross-linking with the Fc region of immunoglobulin E (IgE), FcεRI initiates the activation of MCs through FcεRI-mediated pathways [17,18]. Antigen-specific binding induces the aggregation of IgE fragment–FcεRI complexes, leading to the rapid phosphorylation of the tyrosine kinase LYN. This event facilitates the phosphorylation of the immunoreceptor tyrosine activation motif (ITAM). Once phosphorylated, ITAM recruits the kinases, Src-like kinase (SLK, also called Fyn) and spleen tyrosine kinase (SYK), providing docking sites for SH2 domain–containing proteins and triggering a cascade of signaling events. This cascade ultimately results in calcium influx and alterations in gene transcription levels [19]. Ultimately, the aggregation of FcεRI culminates in the activation and degranulation of MCs, releasing bioactive substances, such as heparin and histamine [20].

In addition, the discovered biphasic reactive receptors also include neurokinin receptors, Mas-related G protein-coupled receptors, Endothelin (ET) receptors, Tropomyosin receptor kinase (Trk) receptors, opioid receptors, and platelet-activating factor [21].

### 3.2 Monophasic Reactive Receptors

Monophasic reactive receptors can be categorized into two distinct activation modes based on the response of MCs after following ligand–receptor interaction. The first activation mode involves MC degranulation exclusively induced by the binding of a specific ligand to a receptor, resulting in the release of preexisting inflammatory mediators. Research on this mode is currently limited, and the complement system has been the primary focus. Within this system, the components C3a and C5a can interact with their respective receptors, C3aR and C5aR, which are expressed on the MC membrane. This interaction facilitates the rapid degranulation of MCs and subsequent release of histamine [22].

Following ligand–receptor binding, the second mode of MC activation involves the synthesis and release of inflammatory factors *de novo* without degranulation. The prototypical receptor involved in this process is c-KIT. Previous studies have established that SCF is an activator of MCs, and its receptor, c-KIT is highly expressed on the surfaces of these cells. Upon binding to SCF, c-KIT undergoes autophosphorylation, initiating a cascade of downstream biological signaling events [23]. The activation of MCs mediated by the c-KIT receptor does not induce rapid degranulation. However, owing to the extensive integration of signaling pathways initiated by c-KIT and FcεRI, SCF binding to c-KIT can enhances the degranulation of MCs and FcεRI-induced release of cytokines simultaneously [24].

Apart from c-KIT, Toll-like receptors (TLRs) play a considerable role in modulating the secondary activation pathway of MCs. Although most TLRs do not trigger MC degranulation, they can stimulate the secretion of various bioactive substances and modulate MC activation mediated by other receptors. Similarly, the activation of MCs by C-type lectin receptors [25] and nucleotide oligomerization domain-like receptor 1 [26] does not result in degranulation but instead induces the release of cytokines and chemokines.

## 4 Secretions of MCs

Chronic low-level inflammation is a key feature of tumors, indicating that MCs play a role in tumor biology [27]. Activated MCs release various bioactive substances, which are categorized into two types: pre-synthesized substances stored in basophilic granules, which constitute an intrinsic feature of MCs, and newly synthesized substances produced after transcriptional activation.

Histamine is a pre-synthesized bioactive compound predominantly derived from MCs. It serves as a critical mediator in allergic and inflammatory responses, facilitating vasodilation, bronchoconstriction, and mucus secretion [28]. In addition, it facilitates tumor proliferation by promoting angiogenesis [29]. In addition to histamine, MCs produce serine proteases, such as tryptase and chymase, which are involved in the proteolytic modification of high-density lipoproteins. Tryptase, released during MC activation, encompasses several isoforms, including tryptase α (encoded by TPSAB1), tryptase β (encoded by TPSAB1 and TPSB2), tryptase δ (encoded by TPSD1), tryptase γ (encoded by TPSG1), and tryptase ε (encoded by PRSS22). These isoforms facilitate angiogenesis, ECM degradation, and decrease adhesion among tumor cells [30,31]. Furthermore, the activation of MCs’ signaling pathways triggers the synthesis and secretion of novel bioactive substances, such as heparinase. Within the TME, MCs secrete heparinase, which interacts with breast cancer cells via the MUC1/estrogen receptor axis, contributing to the maintenance of cancer stem cell properties [32].

Notably, MCs migrating into an intratumoral region are activated in the inflammatory microenvironment and release vascular endothelial growth factor (VEGF), angiopoietin-1, and the chemokines CXCL1 and CXCL8 through degranulation. The receptors of these bioactive substances are distributed in the MCs’ membranes [33]. Therefore, MCs activated in TME further promote the recruitment of MCs by tumor cells through positive feedback [34,35].

## 5 Identification of MCs in the TME

In rodent tissues, MCs are categorized into connective tissue and mucosal MCs according to their anatomical location. Historically, most research on MCs has been conducted using rodent models. However, advancements in research techniques and methodologies have prompted a transition toward utilizing human systems [36]. Currently, the established gold standard for identifying human MCs involves the detection of metachromatic granules within the cytoplasm or the identification of c-KIT and tryptase via immunohistochemistry (IHC) [37]. Based on the type of serine protease they express, mature MCs can be classified into three distinct subsets: chymase-positive MCs (MCCs), tryptase-positive MCs (MCTs), and double protease (Tryptase and chymase)-positive MCs (MCTCs) [38]. Despite these advances, single-molecule detection technologies are limited in accurately determining the spatial localization of MCs and identifying degranulated MCs.

Advancements in sequencing technology have enabled researchers to utilize deconvolution algorithms, such as Cell-type Identification By Estimating Relative Subsets Of RNA Transcripts (CIBERSORT), to estimate the density of MCs within entire tissues [39,40]. Krishnan et al. [41] identified C1q tumor necrosis factor 8 as a characteristic protein of the MCTs subset in prostate cancer. Maimaitiyiming et al. [42] employed machine learning techniques to screen for MC-related genes signatures and were able to identify 13 marker genes across six prostate cancer datasets, collectively termed the MCs marker gene set. Xie et al. [43] applied single-cell RNA sequencing (scRNA-seq) to identify five characteristic MC-related genes (TPSAB1, TPSB2, CPA3, HPGDS, and MS4A2) in human colorectal cancer (CRC), which serve as reliable identifiers of MCs within the TME. Cai et al. [44] established a set of MC-related characteristic genes, including c-KIT, RAB32, CATSPER1, SMYD3, LINC00996, SOCS1, AP2M1, LAT, and HSP90B1. Additionally, Yang et al. [45] developed a predictive model incorporating four quiescent MC-related genes (MS4A2, GATA1, RGS13, and SLC18A2) for the TME of patients with non-small-cell lung cancer. Bao et al. [46] constructed novel molecular subtypes and a neural network prediction model based on an MCs-related gene signature in early-stage lung adenocarcinoma (LUAD). This model accurately predicts immunotherapy response (98.7% accuracy) and reveals associations between the signature, *TP53* mutation, and c-MYC pathway activation, offering novel strategies for immunotherapy and personalized treatment of early-stage LUAD.

Various marker genes for identifying MCs in the TME have been identified, but current techniques, such as single-molecule detection and bulk/single-cell RNA-seq, are limited in their ability to determine MCs’ spatial localization and interactions.

## 6 Functions of MCs in the TME

### 6.1 MCs in Spatial Transcriptomics

With the advent of spatial transcriptomics (ST), a technique that has gained substantial traction, the spatial distribution of MCs within the TME has garnered considerable interest. Unlike traditional sequencing technologies and IHC detection methods used in isolation, ST integrates spatial positioning data and is corroborated by multiplex IHC (mIHC). This integration facilitates a more comprehensive understanding of the localization and functional states of MCs within the TME. Current research on various tumor tissue types indicate a considerable increase in the number of MCs within tumor tissues and their adjacent counterparts [47].

Tumor cells release SCF, which recruits MCs to tumor sites by binding to c-KIT receptors [48]. Mao et al. [49] identified specific MCs in bladder Ewing sarcoma that interact with cancer cells through TNFSF12–TNFRSF12A. Another study [50] discovered that choroid plexus MCs, which rarely express FcεRIα unlike peripheral MCs, disrupt epithelial cells through degranulation, causing tumor-associated hydrocephalus and challenging the notion that the brain lacks immune cells. Xie et al. [43] demonstrated the colocalization of MCs with fibroblasts and endothelial cells. These cells may activate MCs through the KITLG–KIT axis, potentially suppressing tumor progression. Furthermore, Song et al. [51] observed elevated IL1B expression in macrophages within clear cell renal carcinoma samples, and ST analysis revealed the colocalization of VEGFA^+^ MCs with IL1B^+^ macrophages at the tumor–normal interface.

Recently, scRNA-seq has advanced the study of tumor cell composition and identification of malignant cells but struggles to reveal direct interactions and spatial relationships among cell subsets [52]. This limitation is partly due to the low infiltration of MCs in the TME, leading to their underrepresentation [53,54]. Additionally, the high cost and small sample sizes of ST analysis limit its use. ScRNA-seq, bulk RNA-seq, and ST each face challenges in distinguishing MCs in the TME when used alone. To better understand MCs’ roles, integrating IHC or mIHC techniques and using meta-chromatic particles, c-KIT, and tryptase, are recommended [55].

### 6.2 Different Functions of MCs in Different Areas

The infiltration location of MCs is linked to cancer progression. Xia et al. [56] reported that MCs predominantly localize around tumors and to a lesser extent at the invasion margin and within tumors. A method for visualizing TME, “Spatiopath,” shows MCs clustering near cancer cells [57]. In prostate cancer, elevated extratumoral MC density (MCD) correlates with increased recurrence post-surgery [58]. Intratumoral MCs inhibit angiogenesis and tumor growth, whereas peritumoral MCs promote tumor progression [59]. Similarly, in esophageal cancer, MCs at the tumor margin are linked to reduced survival and progression [60].

The role of MCs in breast cancer is debated. Sakalauskaitė et al. [61] found that MCs within tumors correlate with large tumor size and increased lymph node metastases, whereas MCs in the stroma are linked to favorable outcomes and increased lymphangiogenesis [62]. Conversely, a Malaysian study indicated that interstitial MCs have little impact on breast cancer progression [63]. This discrepancy may be attributed to breast cancer molecular types because MCD is notably lower in triple-negative breast cancer than in luminal A and B types [64,65].

### 6.3 Pro-Cancer Mechanisms of MCs

**Figure 1:**
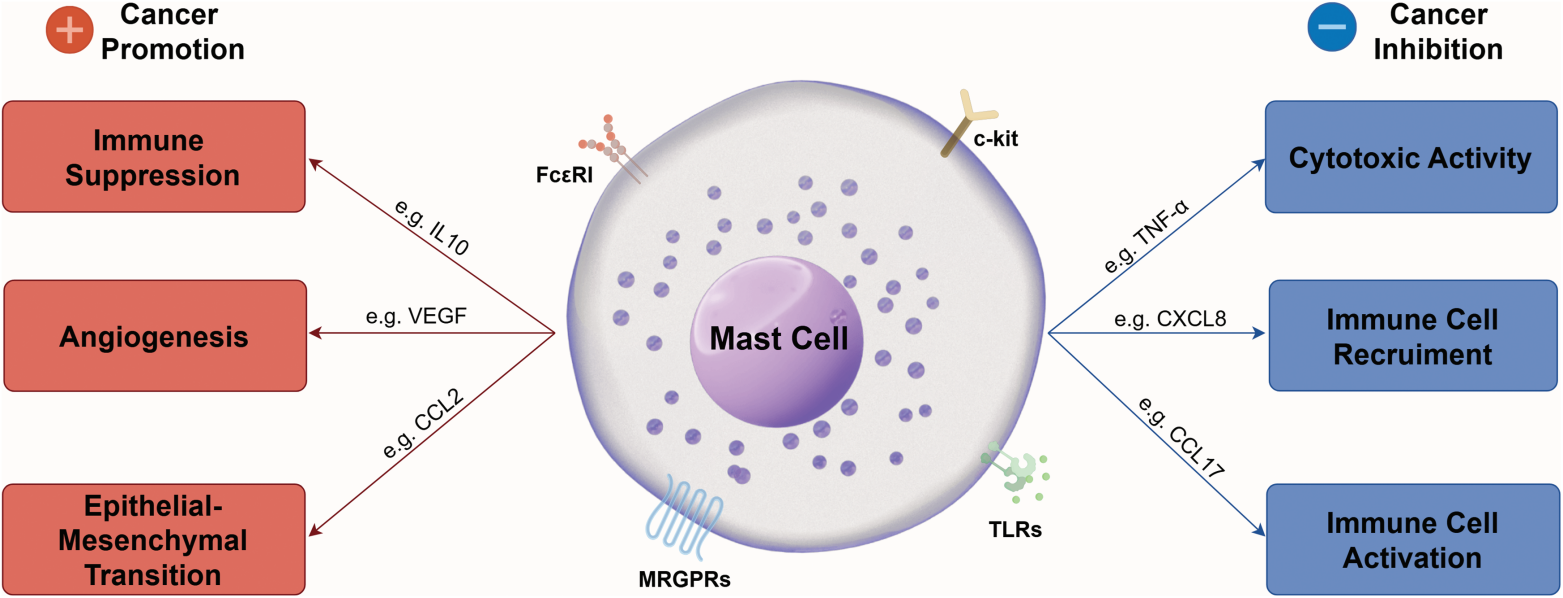
Mechanism of MCs to modulate TME. This figure was generated by Figdraw (ID: PRSIOcd47c)

The mechanism by which MCs modulate the TME is highly complex (Fig. 1). The impact of MC infiltration on tumor progression and patient prognosis varies significantly across tumor types. Elevated MCs infiltration level is associated with tumor progression, recurrence and poorer prognosis, including lung adenocarcinoma [66], cervical cancer [67], bladder cancer [68], thyroid cancer [69,70], CRC [71,72], oral cancer [73], gastric cancer [74–76], pancreatic cancer [77–79], esophageal cancer [80], and breast cancer [32,81].

Furthermore, MCs enhance interactions between estrogen receptors (ERs) and CCL2 within tumors, facilitating bladder cancer invasion through the MC–ER–CCL2 axis, which promotes epithelial-mesenchymal transition (EMT) and matrix metalloproteinase (MMP) activity [82]. Supporting a pro-tumorigenic role, Chang et al. [83] demonstrated suppressed pancreatic ductal adenocarcinoma (PDAC) growth in MC-deficient mice; notably, tumor growth was restored when PDAC cells were transplanted into wild-type mice reconstituted with bone marrow (BM). Similarly, Varma et al. [84] observed increased MC infiltration in recurrent odontogenic keratocyst tissues, further suggesting a pro-invasive function for MCs.

### 6.4 Anti-Cancer Mechanisms of MCs

MCs perform functions beyond promoting cancer progression and thus linking them directly to cancer promoters is overly simplistic. Hempel et al. and Fleischmann et al. [85,86] found that MCs help to prevent the recurrence cases of prostate cancer. Several studies may explain why a higher intratumoral MC density correlates negatively with recurrence. MCs express MHC II for antigen presentation and the costimulatory molecule CD28 [86]. Similar findings were obtained in CRC [87,88], gastric cancer [89], and lung adenocarcinoma [90–92]. These contradictory findings need further investigation. In high-grade serous ovarian cancer, the deadliest gynecologic cancer, MCTCs levels rise after neoadjuvant therapy and serves as a positive prognostic factor [93].

As a component of the innate immune system, the tumor-suppressive effects of MCs are correlated with an increased presence of CD4^+^ T cells and CD15^+^ neutrophils [94]. *In vitro* and *in vivo* studies have demonstrated that MCs inhibit the proliferation of CRC cells and induce apoptosis through the release of various cytokines. This mechanism does not affect normal intestinal epithelial cells [87]. In the context of esophageal adenocarcinoma, the abundance of MCs is associated with low tumor stage and reduced frequency of lymph node metastasis, serving as a prognostic protective factor for patients with lymph node metastasis [95]. Furthermore, Meyer et al. [96] reported that MCs in rodent models exert an inhibitory effect on ovarian tumor growth, suggesting their potential as a novel therapeutic target for ovarian cancer. Given the functional complexity of MCs, research findings are extensive yet inconclusive. We summarize clinically relevant studies on MCs to elucidate the role of MC infiltration levels in solid tumors in Table A1.

### 6.5 Interaction of MCs with Main Components in the TME

#### 6.5.1 Interaction among MCs, T Cells and NK Cells

**Figure 2:**
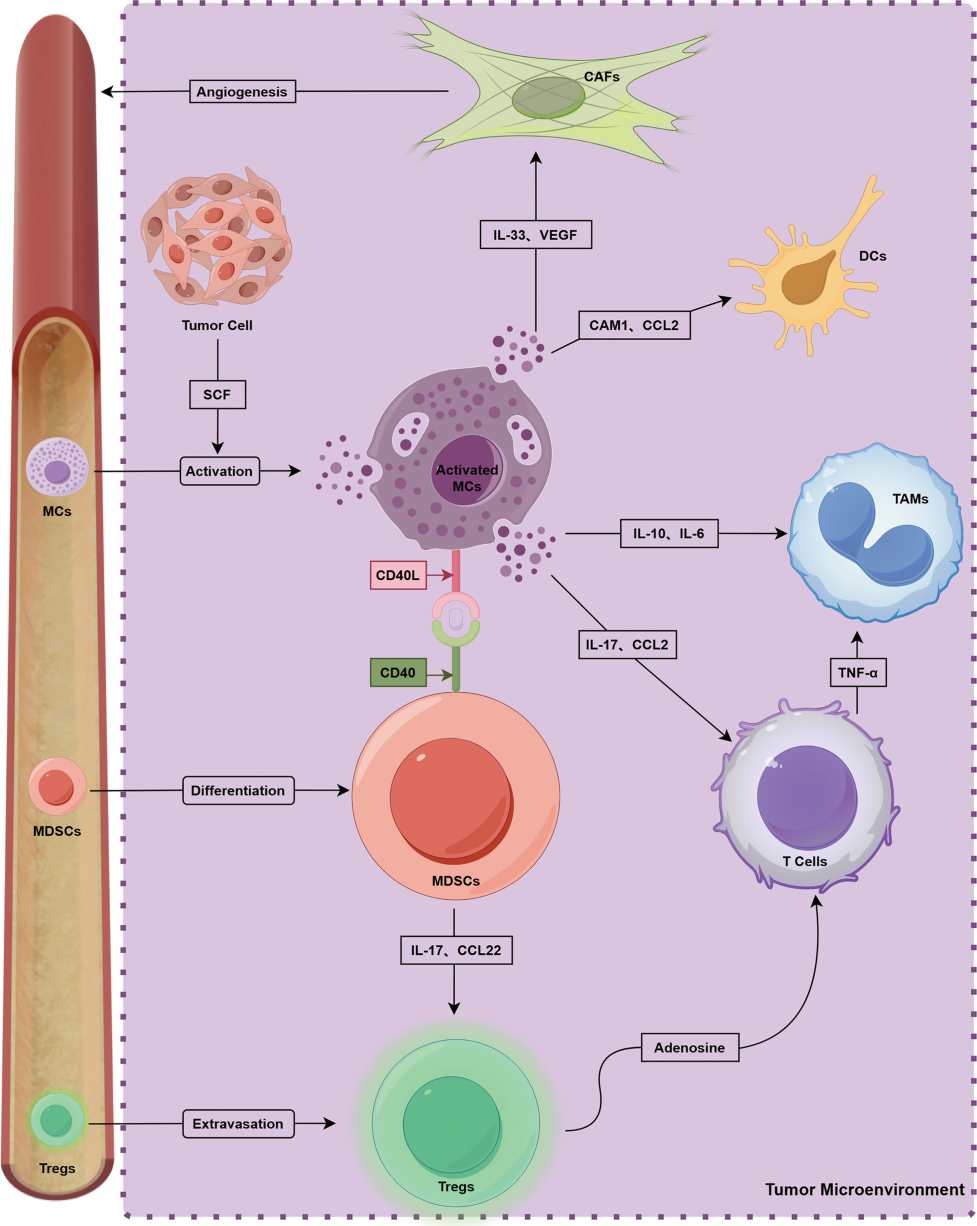
Crosstalk with multiple components and MCs in TME. This figure was generated by Figdraw (ID: ORUUIdd0d8)

MCs play an important role in TME (Fig. 2). T cells and MCs constitute the innate immune system, and various T cell subtypes interact with MCs [29]. Tregs suppress immune response in different types of cancer, and MCs interact with Inducible T-cell co-stimulator (ICOS)^+^ Tregs through IL-33 and IL-2 and promote cancer progression. Additionally, activated MCs release IL-17 and adenosine, boosting Tregs’ proliferation [97]. In CRC, MCs prompt Tregs to enhance inflammation while maintaining their T cell suppression ability [98]. MCs-derived CXCL10 reversely promoted epithelial-mesenchymal transition and induced immunosuppressive TME of PDAC by recruiting CXCR3^+^ Tregs [99].

Various studies on tumor immunotherapy highlight the immune system’s capability to combat cancer, with CD8^+^ T cells being crucial for antitumor immunity [100]. In esophageal cancer, IL17^+^ MCs attract CD8^+^ T cells and tumor-associated macrophages (TAMs) to inhibit cancer progression [101]. Similarly, in lung adenocarcinoma, certain MCs secrete CCL2 to recruit CCR2^+^ cytotoxic T cells, enhancing Tyrosine kinase inhibitors (TKIs) treatment [102]. However, MCs can also suppress T cell antitumor activity. In pancreatic cancer, anti-OX40 treatment combined with MCs depletion increased CD8^+^ T cells infiltration [103]. MCs expressing galectin-9 inhibit CD8^+^ T cell immunity [70], and increased intratumoral MCs promote immune suppression and gastric cancer progression via the TNF-α-PD-L1 pathway [76]. In mice, MC deficiency inhibits CRC development while promoting CD8^+^ T cell infiltration [104].

The cross-talk between MCs and natural killer (NK) cells mainly acts on tumor surveillance and regression. NK cells rarely infiltrate tumors because of immunosuppressive microenvironments, although high-density MCs can release cytokines, such as CXCL8, CCL3, IFN, and histamine, which can recruit NK cells to suppress tumor progression [105].

#### 6.5.2 Interaction between MCs and Myeloid-Derived Suppressor Cells

Myeloid-derived suppressor cells (MDSCs) play a crucial role in immunosuppression [106]. According to Jachetti et al. [107], MCs and MDSCs contribute to tumor pathogenesis through interactions involving the CD40L–CD40 axis, and MDSCs activate MCs by secreting TNF-α, IL-6, and other factors. Furthermore, IL17-positive MCs have the capacity to recruit MDSCs, thereby facilitating cancer progression [108]. Notably, a feedback loop appears to exist among MCs, MDSCs, and Tregs. This loop allows for the concurrent development of the TME and immunosuppression. MDSC-recruited MCs increase the levels of chemokines CCL17 and CCL22 through interactions with CCL2, which subsequently enhances the immunosuppressive function of Tregs [8].

#### 6.5.3 Interaction between MCs and TAMs

Intercellular communication between MCs and TAMs constitutes an essential element in the regulation of tumor immunity. This interaction facilitates the development and progression of CRC [109]. Specifically, the activation of MCs via IL-33 enhances cancer progression and elevates microvessel density (MVD) by recruiting TAMs [110]. Liu et al. [111] demonstrated that MCs activate the IFN and NF-κB signaling pathways of bladder cancer cells within a co-culture system. This process increases the secretion of CCL2 and IL-13 by bladder cancer cells, thereby promoting the polarization of monocytes into TAMs.

#### 6.5.4 Interaction between MCs, Cancer-Associated Fibroblasts, and Adipocytes

The dynamic interplay between tumor cells and their surrounding ECM is widely recognized as a crucial factor in the formation, progression, metastasis, and development of drug resistance in solid tumors. However, the relationship between the stroma and tumor-infiltrating lymphocytes remains unclear. Nonetheless, substantial evidence indicates that the composition of the stroma considerably influences anti-tumor immunity and the responsiveness to immunotherapy [112].

Cancer-associated fibroblasts (CAFs) play a crucial role in the TME, influencing ECM remodeling, tumor growth, drug resistance, and metastasis [113]. MCs regulate fibroblasts within the TME, promoting myofibroblast differentiation in breast cancer through tryptase activity [114]. SAMD14 acts as a tumor suppressor, and its overexpression modifies MCs, leading to increased CAF deposition in prostate cancer [115]. Additionally, exosome-associated IL33 activates MCs in pancreatic cancer, which then converts fibroblasts into inflammatory CAFs. However, this process can be inhibited by BAG6 overexpression [116].

Apart from CAFs, adipocytes are stromal cells too. Adipose tissue serves as a reservoir for MC progenitors, potentially recruiting MCs to the TME [117]. Additionally, MCs from adipose tissues can target and induce apoptosis in breast cancer cells [118].

#### 6.5.5 MCs in Tumor Angiogenesis

Tumor angiogenesis, driven by endothelial cells, is crucial for tumor growth and spread by supplying oxygen and nutrients through new blood vessels [119]. A link between MCD and MVD in tumors has been established [120], and MCD had been positively correlated with MVD in oral squamous cell carcinoma [121], lung cancer [122], CRC [123], and gastric cancer [124,125]. Low proliferative activity and MVD were observed in melanoma cells inoculated in MCs-deficient mice but normalized after BM transplantation from normal mice. MCs enhance angiogenesis and tumor growth in cutaneous melanoma through the autocrine/paracrine signaling of SCF, CCL5, CCL11, and CCL18 [126–129].

Chymase and tryptase, which are key serine proteases from MCs, are closely linked to angiogenesis [130–132]. High tryptase levels correlate with increased MC infiltration and MVD [133]. Tumor-derived exosomes can deliver SCFs to MCs, influencing tryptase release and vascular endothelial growth [134]. Tryptase serves as a prognostic marker and is involved in angiogenesis across various types of cancer by regulating endothelial cell PAR-2 expression [89,135]. This evidence suggests that MCs play a role in promoting tumor angiogenesis and metastasis.

Some metabolites affect the function of MCs. For example, in cholangiocarcinoma, bile induces MCs to secrete platelet-derived growth factor B and angiopoietin 1/2, enhancing angiogenesis in cholangiocarcinoma [136].

## 7 Targeted Therapy Strategies for MCs

### 7.1 Target the c-KIT Signal

There are many targeted therapy strategies for MC (Table 1), among which SCF/c-KIT is crucial for MC’s development and function, and TKIs, such as imatinib and nilotinib, can target MCs in some conditions, such as mastocytosis and allergies, by inhibiting the binding of SCF to c-KIT receptors [21]. Although pan-TKIs have been successfully used in cancer treatment, their ability to target MCs is limited because of off-target effects [6]. To address this limitation, barzolvolimab, which is a c-KIT-specific monoclonal antibody, has been developed. However, it has been tested only in neurofibromatosis and solid tumors (NCT02642016 and NCT02642016) [137–139]. Unfortunately, until now, all clinical trials of barzolvolimab for the treatment of solid tumors have not had published results.

**Table 1:** Therapeutic targets of MCs

Reference	Drug	Therapy Strategies	Target	Disease, Animal or Cell Model
[137,140]	Barzolvolimab and iSCK03	Target the c-KIT signal	c-KIT	Neurofibromatosis and solid tumors
[141]	Sunitinib and Imatinib	Target the c-KIT signal	Tyrosine kinase	Melanoma
[69,142,143,145]	Cromolyn	Stabilizing the MCs membrane	GSK-3β	Colon cancer, Thyroid cancer and Breast cancer
[144]	Clarithromycin	Stabilizing MC’s membrane	MRGPRX2	Breast cancer
[144]	Human HER2/*neu*-specific IgE and IL-17-neutralizing antibody	Stabilizing MC’s membrane	FcεRI	Breast cancer and gastric cancer
[155]	Tranilast	Target MCs downstream and stabilization of the cell membrane	TGF-β and IgE	Gastric cancer
[157–160]	Pam3CSK4, LPS and Imiquimod	Stimulate anti-tumor immunity mediated by MCs	TLR2, 4 and 7	Melanoma and Actinic keratosis

Note: MCs, Mast cells; c-KIT, Mast/stem cell growth factor receptor Kit; GSK-3β, Glycogen synthase kinase-3 beta; MRGPRX2, Mas-related G protein-coupled receptor X2; FcεRI, Fc epsilon RI; IgE, Immunoglobulin E; TGF-β, Transforming growth factor beta; TLR, Toll-like receptor; IL-17, Interleukin-17; HER2, Human epidermal growth factor receptor 2; LPS, Lipopolysaccharide.

In a preclinical study, Yang et al. [140] discovered that the c-KIT inhibitor iSCK03 boosts apoptosis signaling in breast cancer, enhancing antitumor effects. Additionally, combining anti-PD-1 therapy with sunitinib or imatinib leads to MC depletion and complete tumor regression [141].

### 7.2 Stabilizing MC Membrane

Stabilizing MC membrane to inhibit MC activation and degranulation is a common therapeutic approach in allergy treatment. In this approach, clarithromycin, cromolyn, and ketotifen are commonly used [5], which have shown promise in preclinical cancer models. For example, the effectiveness of cromolyn in inhibiting tumor growth has been demonstrated in colon [142], thyroid [69], and breast cancer models [143–145]. In a phase II clinical trial (NCT05076682), cromolyn combined with anti-PD1 drugs demonstrated a synergistic effect in triple-negative breast cancer (TNBC) by targeting MCs, marking the only clinical trial focused on specific MC subset, antigen-presenting MCs (APMCs) [145]. This clinical trial demonstrated that combining cromolyn with anti-PD1 therapy in patients with metastatic TNBC refractory to prior immunotherapy achieved an objective response rate (ORR) of 50% with a favorable safety profile, offering a novel strategy for optimizing immunotherapy by targeting APMCs.

The application of IgE antibodies to enhance antitumor response has been proposed because they can inhibits MCs’ degranulation [146,147]. IgE is crucial for MCs activation, and monoclonal antibodies (mAbs), such as omalizumab, ligelizumab, quilizumab, and UB-221 block IgE from binding to FcεR, thus preventing MC activation [148]. *In vitro*, humanized monoclonal anti-HER-2/neu IgE and anti-CD20 IgE have been shown to inhibit MCs’ degranulation and tumor growth [149].

In addition to traditional mAbs, recombinant proteins, such as sdab-026, E2_79 and bi53_79, have been reported to have anti-IgE activity. Furthermore, Gunjigake et al. [150] reported that a recombinant IL-17-neutralizing antibody can inhibit MCs’ degranulation. These recombinant proteins are more productive and stable than mAbs. The route of administration is easier than that of mAbs and can be used through the mucous membranes.

Complement C3a-derived peptides, C3a7 and C3a9, may interact with the β chain of FcεR on the membranes of MCs, potentially decreasing the likelihood of IgE binding to FcεR and consequently influencing signal transduction [151–153]. However, this hypothesis has not been thoroughly investigated, and the precise function of these interactions remains unclear.

### 7.3 Target MC Downstream

The direct regulation of bioactive substances secreted by MCs represents an effective strategy for modulating MCs activation [154]. Tryptase inhibitors, such as triniste, nafamostat mesylate, and gabexate mesylate has demonstrated antitumor efficacy in preclinical studies involving multibell solid tumors, either as monotherapies or in combination with other therapeutic agents [3]. Nakamura et al. [155] revealed that triniste inhibits the infiltration of MCs and TAMs within tumors while simultaneously enhancing infiltration by CD8^+^ T cells. Furthermore, Ma et al. [156] conducted an analysis of the three-dimensional structure of tryptase isoforms, leading to the identification and validation of the tryptase β-selective inhibitor compound 22978-61-6 (3,5-dichlorophenyl-1-carboxamide hydrochloride).

### 7.4 Stimulate Anti-Tumor Immunity Mediated by MCs

While substantial evidence supports the tumor-promoting roles of MCs, recent studies suggest that MCs, as integral components of the innate immune system, may also exhibit antitumor immune functions. Activation of MCs via TLRs leads to the secretion of cytokines with both direct and indirect antitumor activities. For example, in the B16.F10 murine melanoma model, TLR2 agonists stimulate the release of MC-derived interleukin-6 and CCL3, which directly inhibit tumor growth and indirectly facilitate the recruitment of natural killer cells (NKs) [157]. Although the potential for systemic toxicity necessitates thorough evaluation, these findings advocates for the investigation of TLR2 agonists as localized immunomodulators or in conjunction with NK cell-based therapies.

TLR4, the receptor specifically recognizing lipopolysaccharide (LPS), stimulates MCs to secrete CXCL10, thereby facilitating the recruitment of T cells in melanoma models. This position intratumoral LPS injection as a potential adjunctive strategy to enhance clinical anti-CTLA-4 therapy [158]. Despite its promise, successful clinical translation requires the development of safer synthetic TLR4 agonists tailored for human application, optimization of injection protocols, and validation of efficacy through clinical trials. Moreover, the pretreatment of MCs with the TLR7 agonist imiquimod has been shown to enhance the expression of co-stimulatory and activating molecules in dendritic cells (DCs), thereby improving the efficacy of targeting vaccines to DCs [159]. This approach presents a viable strategy for augmenting current DC vaccine. Additionally, imiquimod induces the release of CCL2 from MCs, facilitating the recruitment of plasmacytoid DCs to tumor sites and thereby promoting antitumor immunity [160]. Given imiquimod’s approval for the treatment of basal cell carcinoma and its established clinical efficacy, its repurposing, either as a monotherapy or in conjunction with DC vaccines, to activate MCs and modify TME holds considerable translational promise. However, the complexity of MC functions, alongside their potential protumorigenic effects, highlights the necessity for meticulous patient stratification and the identification of relevant biomarkers.

## 8 Conclusion

The diverse functions of MCs within the TME, along with recent studies investigating MCs as novel targets, have increasingly been discussed in the context of tumor immunotherapy. However, the overall potential of MC-targeted immunotherapy remains uncertain, which may be attributed to ambiguities in subset analysis of MCs and technical limitations in accurately identifying and characterizing these cells within the TME.

MCs play crucial roles in the TME, prompting the need to develop innovative approaches to differentiate MC subsets through the evaluation of new markers. Future research should emphasize the precise labeling of MC-specific subtypes, as certain subsets may represent promising targets for tumor immunotherapy. Nevertheless, extensive investigation is required before MC-targeted therapies can be effectively implemented. Additionally, therapeutic strategies designed to specifically modulate the functions of MC subsets must be carefully engineered to minimize off-target or contradictory effects.

The functional roles of MCs should also be explored in combination with other immunotherapeutic strategies to evaluate potential synergies between different treatment agents. While numerous *in vivo* and *in vitro* studies support this direction, comprehensive clinical trials focusing on MCs are essential to clearly establish the clinical relevance and therapeutic benefits of MC-targeted interventions in cancer patients.

It is important to note, however, that phenotypic and functional differences—including activation pathways—between murine and human MCs may hinder the translation of findings from animal models to clinical applications.

## Data Availability

The data that support the findings of this study are available from the corresponding authors upon reasonable request.

## Abbreviations

BM: Bone Marrow
CAFs: Cancer-associated fibroblasts
CMA1: Mast cell protease-1
CTMCs: Connective tissue mast cells
CTRP8: C1q tumor necrosis factor 8
DCs: Dendritic cells
ECM: Extracellular matrix
ERs: Estrogen receptors
ET: Endothelin
Trk: Tropomyosin receptor kinase
FGF: Fibroblast growth factor
FcεRI: Fc epsilon RI
HNSCC: Head and neck squamous cell carcinoma
HSCs: Hematopoietic stem cells
IgE: Immunoglobulin E
ICAFs: Inflammatory cancer-associated fibroblasts
ICOS: Inducible T-cell co-stimulator
ITAM: Immunoreceptor tyrosine activation motif
LTB4: Leukotriene B4
LTs: Leukotrienes
mAbs: Monoclonal antibodies
MCC: Chymase-positive mast cell
MCD: Mast cell density
MCMGs: MCs marker gene sets
MCs: Mast cells
MCT: Tryptase-positive mast cell
mIHC: Multiplex immunohistochemistry
MIRR: Multi-chain immune recognition receptor
MMCs: Mucosal mast cells
MCTCs: (Tryptase and chymase)-positive mast cells
MRGPRs: Mas-related G protein-coupled receptors
MDSCs: Myeloid-derived suppressor cells
MVD: Microvessel density
NKRs: Neurokinins
NKs: Natural killer cells
NOD1: Nucleotide oligomerization domain-like receptor 1
NSCLC: Non-small-cell lung carcinoma
OSCC: Oral squamous cell carcinoma
PAF: Platelet-activating factor
PDGF-B: Platelet-derived growth factor B
PGE2: Prostaglandin E2
PGs: Prostaglandins
RFS: Recurrence-free survival
SCF: Stem cell factor
SCGF: Stem cell growth factor receptor
ST: Spatial transcriptomics
SLK: Src-like kinase
SYK: Spleen tyrosine kinase
TAMs: Tumor-associated macrophages
TCGA: The Cancer Genome Atlas
TLRs: Toll-like receptors
TME: Tumor microenvironment
TNBC: Triple-negative breast cancer
TNF-α: Tumor necrosis factor-α
TKIs: Tyrosine kinase inhibitors
VEGF: Vascular endothelial growth factor

## Appendix A

**Table A1:** Role of MCs in the outcome of different solid tumors

Reference	Prognosis/Recurrence	Methods of MCs identification	Disease	Stage
[67]	Negative	CIBERSORT and IHC (Tryptase)	Cervical Carcinoma	All TNM stages
[71,72]	Negative	CIBERSORT and IHC (Tryptase)	Colorectal cancer	All TNM stages
[87]	Positive	IHC (Tryptase α/β1)	Colorectal cancer	All TNM stages
[88]	Positive	IHC (Tryptase/Chymase)	Colorectal cancer	Stage I and IV
[95]	Positive	IHC (CD117)	Esophageal adenocarcinoma	All UICC stages
[80]	Negative	CIBERSORT/IHC (Tryptase)	Esophageal carcinoma	All TNM stages
[73,75]	Negative	IHC (Tryptase)	Gastric cancer	All TNM stages
[76]	Negative	Flow cytometric	Gastric cancer	All TNM stages
[89]	Positive	IHC (Tryptase)	Gastric cancer	Stage I
[93]	Positive	Hematologic Stain	High grade serous ovarian cancer	Stage Ⅲ and IV
[90]	Positive	IHC (CD117)	Lung Adenocarcinoma	Stage I and Ⅲ
[68]	Negative	CIBERSORT/IHC (Tryptase)	Muscle-invasive bladder cancer	All TNM stages
[91]	Positive	Flow Cytometry/IHC (Tryptase)	Non-Small Cell Lung Cancer	Stage I Ⅱ and Ⅲ
[92]	Positive	IHC (Tryptase/Chymase)	Non-Small Cell Lung Cancer	Stage I, II, Ⅱ and Ⅲa
[84]	Negative	IHC (CD117)	Odontogenic Keratocysts	Not applicable
[73]	Negative	CIBERSORT/IHC (CD117)	Oral cancer	Non report
[96]	Positive	Toluidine blue	Ovarian Cancer	Non report
[79]	Negative	IHC (Tryptase/CD117)	Pancreatic ductal adenocarcinoma	All TNM stages
[83]	Negative	Toluidine blue	Pancreatic ductal adenocarcinoma	All TNM stages
[94]	Positive	IHC (CD117)	Pancreatic Neuroendocrine Neoplasms	All TNM stages
[69]	Negative	IHC (Tryptase)	Papillary thyroid carcinoma	All TNM stages
[70]	Negative	IHC (CD45/CD117/FcεRI)	Papillary thyroid carcinoma	Stage Ⅲ and IV
[85]	Positive	IHC (Tryptase)	Prostate Cancer	All TNM stages
[86]	Positive	IHC (CD117)	Prostate Cancer	All T stages

Note: MCs, Mast cell; IHC, Immunohistochemistry; FcεRI, Fc epsilon RI; TNM, Tumor Node Metastasis; UICC, Union for International Cancer Control; CIBERSORT, Cell-type Identification by Estimating Relative Subsets of RNA Transcripts.
